# Effectiveness and tolerability of repetitive transcranial magnetic stimulation for preventive treatment of episodic migraine: a single-centre, randomised, double-blind, sham-controlled phase 2 trial (Magnet-EM)

**DOI:** 10.1186/s13063-020-04832-y

**Published:** 2020-11-11

**Authors:** Nabil Izzaatie Mohamad Safiai, Nur Ain Amir, Hamidon Basri, Liyana Najwa Inche Mat, Fan Kee Hoo, Abdul Hanif Khan Yusof Khan, Wei Chao Loh, Peck Kee Chia, Vasudevan Ramachandran, Hazwan Mat Din, Intan Nureslyna Samsudin, Aaron Fernandez, Mohd Hazmi Mohamed, Siew Mooi Ching, Hasnur Zaman Hashim, Wan Aliaa Wan Sulaiman

**Affiliations:** 1 Department of Medicine, Faculty of Medicine and Health Sciences, Universiti Putra Malaysia (UPM), 43400 Serdang, Selangor Malaysia; 2 Malaysian Research Institute on Ageing, Universiti Putra Malaysia (UPM), 43400 Serdang, Selangor Malaysia; 3 Department of Pathology, Faculty of Medicine and Health Sciences, Universiti Putra Malaysia (UPM), 43400 Serdang, Selangor Malaysia; 4 Department of Psychiatry, Faculty of Medicine and Health Sciences, Universiti Putra Malaysia (UPM), 43400 Serdang, Selangor Malaysia; 5 Department of ORL-HNS, Faculty of Medicine and Health Sciences, Universiti Putra Malaysia (UPM), 43400 Serdang, Selangor Malaysia; 6 Department of Family Medicine, Faculty of Medicine and Health Sciences, Universiti Putra Malaysia (UPM), 43400 Serdang, Selangor Malaysia; 7 Neurology services, Columbia Asia Hospital-Klang, Jalan Mahkota 1/ KU2, Mutiara Bukit Raja 2, 41050 Klang, Selangor Malaysia

**Keywords:** Migraine, Repetitive transcranial magnetic stimulation (TMS), Randomised controlled trial

## Abstract

**Background:**

This is a phase II randomised, double-blind, sham-controlled trial to evaluate the effectiveness and tolerability of repetitive transcranial magnetic stimulation for preventive treatment of episodic migraine amongst migraine subjects.

**Methods:**

Subjects age 18 to 60 years will undergo a baseline evaluation to establish the diagnosis of migraine based on the International Classification of Headache Disorder 3rd Edition (ICHD-3). Those who fulfil the ICHD-3 criteria for episodic migraine and compliant to the headache diary during a month run-in period will be enrolled. A total of 76 subjects will be randomised to receive either transcranial magnetic stimulation or sham stimulation for 5 sessions within 2 weeks duration. Follow-up sessions will be conducted monthly for three consecutive months. Prior to treatment, subjects will be required to fill up questionnaires and undergo few procedures such as electroencephalography, transcranial Doppler ultrasound and biochemical analysis for serum serotonin, serum calcitonin gene-related peptide and serum beta-endorphin. These procedures will be repeated at month 3 after receiving the last treatment. The primary outcome measure of this study is the difference in mean monthly migraine days at baseline and at months 1, 2 and 3 after treatment sessions.

**Discussion:**

Following evidence from previous studies showing restoration of dorsolateral prefrontal cortex (DLPFC) activation to almost normal level, the rTMS intervention will target left DLPFC in this study. An intermediate duration of treatment sessions is selected for this study. It is set to five treatment sessions given within 2 weeks duration.

**Trial registration:**

ClinicalTrials.gov NCT03556722. Registered on 14 June 2018

## Administrative information

**Table:** 

Title {1}	Effectiveness and Tolerability of Repetitive Transcranial Magnetic Stimulation for Preventive Treatment of Episodic Migraine: A Single Centre, Randomised, Double-Blind, Sham-Controlled Phase 2 Trial (MAGNET-EM).
Trial registration {2a and 2b}	ClinicalTrials.gov NCT03556722.
Protocol version {3}	01/10/2019 Version 4.
Funding {4}	This work is supported by Research Management Centre, Universiti Putra Malaysia (Grant Number GPB/2017/9585500).
Author details {5a}	1. Department of Medicine, Faculty of Medicine and Health Sciences, Universiti Putra Malaysia, 43400 UPM Serdang, Selangor, Malaysia. 2. Malaysian Research Institute on Ageing, Universiti Putra Malaysia, 43400 UPM Serdang, Selangor, Malaysia. 3. Department of Pathology, Faculty of Medicine and Health Sciences, Universiti Putra Malaysia, 43400 UPM Serdang, Selangor, Malaysia. 4. Department of Psychiatry, Faculty of Medicine and Health Sciences, Universiti Putra Malaysia, 43400 UPM Serdang, Selangor, Malaysia. 5. Department of Surgery, Faculty of Medicine and Health Sciences, Universiti Putra Malaysia, 43400 UPM Serdang, Selangor, Malaysia. 6. Department of Family Medicine, Faculty of Medicine and Health Sciences, Universiti Putra Malaysia, 43400 UPM Serdang, Selangor, Malaysia. 7. Avisena Specialist Hospital, Medical Department Jalan Ikhtisas, Seksyen 14, 40000 Shah Alam, Selangor, Malaysia.
Name and contact information for the trial sponsor {5b}	Professor Dr Mohd Adzir Mahdi Telephone No.: (+60) 397691610 Fax No.:(+60) 397691610 Email: dir.rmc@upm.edu.my
Role of sponsor {5c}	Providing fund, implement quality control and quality assurance, monitoring the progress of the research trial, review the recorded data and ensuring the researchers comply to the study protocol.

## Introduction

### Background and rationale {6a}

Migraine is a common neurological problem encompassing about 11% global prevalence around the globe [1]. One-year prevalence for migraine in the Asia-Pacific Region is 9.1% (1.5–22.8%) which is relatively consistent throughout the region [2]. Migraine is a neurological problem most commonly begins at a young age during the first three decades of life and peaks at puberty which is around the age of 12 and 15 for boys and girls, respectively [3].

Transcranial magnetic stimulation (TMS) is a safe non-invasive neuromodulation and effective non-pharmacological migraine treatment. TMS is given using a device that delivers a predetermined level of magnetic pulses to the scalp. In repetitive TMS (rTMS), a train of TMS pulses, like those given in single-pulse TMS (sTMS), is applied at frequencies of 1–50 Hz. Low-frequency rTMS (1 Hz) has been demonstrated to inhibit cortical excitability, whereas high-frequency stimulation (5–20 Hz) may increase cortical excitability [4].

Since its introduction in 1985 [5], many studies were done to determine the safety and efficacy of TMS. Prior to the first rTMS approval by the Food and Drug Administration (FDA) in 2008 for major depressive disorder, earlier studies had reported that the most common side effects were mild such as headache, neck ache and drowsiness [6, 7]. A recent retrospective study done in migraine patient with comorbid depression found that rTMS is well-tolerated by the patients, and it was able to reduce headache frequency, headache severity and depression rating scale [8].

In a recent systematic review [9], only 3 migraine studies using rTMS were graded as a high-quality study with low risk of overall bias. In one of the study, the researchers did a randomised, double-blind clinical trial on 11 chronic migraine patients using 90% stimulation intensity of resting motor threshold (RMT) at 20 Hz frequency [10]. The left dorsolateral prefrontal cortex (DLPFC) was targeted, and a total of 400 pulses were given in each session. The result of the study showed rTMS was safe with no reported side effects and effective to reduce migraine attack, number of abortive pills and headache index.

Another study included in the review was on chronic migraine targeting the left DPFLC at 10 Hz frequency using 100% RMT [11]. Twenty-three treatment sessions were delivered within 8 weeks. A total number of 1600 pulses were given in each session with 30-s inter-train interval (ITI). However, the outcome showed that rTMS did not reduce headache days in chronic migraine. Besides, this study reported that 78% of patients in the rTMS group had pain at the site of treatment or onset of headache or worsening of headache during rTMS treatment, while in the sham group, only 33% reported the same complaint.

In a different study, a meta-analysis done across five randomised trial using high-frequency TMS in migraine had shown positive results. However, there are many variabilities across the studies and many uncertainties regarding the extent of the efficacy of rTMS specifically the information on the doses, location of stimulation and number of sessions [7]. Considering all of these factors and both positive and negative results of previous studies, we have developed a randomised control trial to investigate whether rTMS is effective as a preventive therapy in treating episodic migraine.

### Objectives {7}

The main objective of this study is to evaluate the efficacy of rTMS as a preventive treatment of episodic migraine subjects. We hypothesise that rTMS is an effective prophylaxis for episodic migraine.

### Trial design {8}

This study is a single-centre, randomised, double-blind trial comparing sham and active transcranial magnetic stimulation.

## Methods: participants, interventions and outcomes

### Study setting {9}

This trial is currently undergoing in the Headache Research Clinic, Neurophysiology Laboratory, in the Faculty of Medicine and Health Sciences, Universiti Putra Malaysia, 43400 Seri Kembangan, Selangor, Malaysia.

### Eligibility criteria {10}

The following are the inclusion criteria:

1. Males or females aged 18 to 60 years of age
2. Subjects fulfilling the criteria for episodic migraine as per the third edition of the International Headache Society (ICHD-3) for at least 1 year
3. Frequency of migraine attacks 2–8 times per month with less than 15 headache days per month for at least 3 months prior to screening
4. Demonstrated compliance with the headache diary during the run-in period by entry of headache data on a minimum of 24/30 days (80% compliance)
5. A signed and dated informed consent document indicating that the subject has been informed of all pertinent aspects of the study including any known and potential risks and available alternative treatments

The following are the exclusion criteria:

1. Patients with a previous history of rTMS treatment
2. Onset of headache at more than 50 years old [12]
3. Headache with red flag symptoms that may suggest organic secondary headaches
4. Pregnant or lactating women
5. Patients with contraindications to TMS such as metallic implant and pacemaker based on the Screening 13-item Questionnaire for rTMS candidate
6. Patients with medical conditions such severe hypertension, infections, malignancy, cardiovascular and cerebrovascular disease, epilepsy, degenerative central nervous system diseases, renal failure, hepatic failure, bleeding diathesis and any psychiatric patients who have serious mental illness

### Who will take informed consent? {26a}

The principal investigator and any research members who had been delegated the task to request informed consent from the participants by the principal investigator. The researchers must have a valid Good Clinical Practice (GCP) certificate awarded by the National Pharmaceutical Regulatory Authorities (Malaysia).

### Additional consent provisions for collection and use of participant data and biological specimens {26b}

Any collection of data or biological specimens for future use will need to request a new ethical approval from the institutional review board and new consent from participants.

## Interventions

### Explanation for the choice of comparators {6b}

The sham stimulation will be given using a sham coil in the same manner as rTMS. The sham coil is a Magstim Rapid-2 (Whitland, Walsh, UK), 70mm Double Air Film Sham Coil. The Magstim Air Film sham coil is identical in all but stimulation output. Patients assigned to the sham coil will receive exactly the same number of total sessions as those who undergo active rTMS stimulation.

### Intervention description {11a}

The magnetic stimulation will be given using Magstim Rapid-2 (Whitland, Walsh, UK), 70mm Double Air Film Coil. The magnetic stimulator will be placed antero-posteriorly parallel to the midline on the left dorsolateral prefrontal cortex [10] corresponding to the hot spot of the right abductor digiti minimi (7 cm lateral and 5 cm anterior to the inter-aural line). The motor threshold will be determined prior to the first session of the intervention at the hot spot of the right abductor digiti minimi. Motor threshold is defined as the minimum stimulus intensity able to elicit 5 or more motor-evoked potentials of 50 IV out of 10 consecutive stimuli.

Only 80% of the motor threshold will be used to stimulate the DLPFC in this study. Each session of rTMS will consist of 2000 pulses given in 40 cycles. Each cycle duration lasts for 2.5 s and contains only 50 pulses. The stimulation frequency is set at 20 Hz, and the duration of the inter-train interval will be 25 s long. The treatment sessions are designed to be delivered within 2 weeks, in which the first three sessions will be done consecutively during the first week and the last two sessions will be given consecutively in the second week. In total, participants will receive 10,000 cumulative pulses in the span of 2 weeks. Adverse events will be monitored during the stimulation period until 4 weeks after receiving the last treatment session.

### Criteria for discontinuing or modifying allocated interventions {11b}

There will be no modification allowed to the allocated intervention in this trial. The criteria for discontinuing allocated interventions are as follows:

1. Pregnancy
2. Subject’s withdrawal

However, any data collected up to the time of discontinuation will still be used for the study.

### Strategies to improve adherence to interventions {11c}

A few methods to improve compliance such as check-ups, pamphlets and consultation will be applied. Every patient will be requested to choose the treatment sessions and schedule appointment depending on their availability and the available slot (to prevent redundancy of participants). The research team will remind the participants regarding their upcoming appointment through phone call or messages.

### Relevant concomitant care permitted or prohibited during the trial {11d}

Any concomitant care either for migraine or any other illnesses is allowed throughout this study.

### Provisions for post-trial care {30}

All patients are entitled to medical insurance coverage in this study.

### Outcomes {12}

The primary outcome measure of this study is the changes in mean monthly migraine days during a month before randomisation and months 1, 2 and 3 after treatment sessions. The secondary outcome measures are differences in mean monthly migraine attacks, proportion of subjects with at least a 50% reduction from baseline in mean monthly migraine days, change from baseline in mean monthly pain intensity of migraine attacks, frequency and severity of adverse events in response to rTMS, the pattern changes in electroencephalography (EEG) and transcranial Doppler sonography (TCD) at baseline and 3 months after the last treatment session, serum serotonin, serum calcitonin gene-related peptide (CGRP) and serum beta-endorphin level changes at baseline and 3 months after the last treatment session, Migraine-Specific Questionnaire version 2 (MSQv2.1) at baseline and 3 months after the last treatment session, Depression Anxiety and Stress 21 Scale (DASS21) at baseline and 3 months after the last treatment session, European Quality of Life 5 Dimension (EQ-5D) at baseline and 3 months after the last treatment session, Migraine Disability Index (MIDAS) at baseline and 3 months after the last treatment session, Pittsburgh Sleep Quality Index (PSQI) at baseline and 3 months after the last treatment session, Global Physical Activity Questionnaire (GPAQ) at baseline and 3 months after the last treatment session, Food Frequency Questionnaires (FFQ) at baseline, and satisfaction measures of efficacy, tolerability, safety and expectations of rTMS amongst the participants at 3 months after the last treatment session.

### Participant timeline {13}

**Fig. 1:**
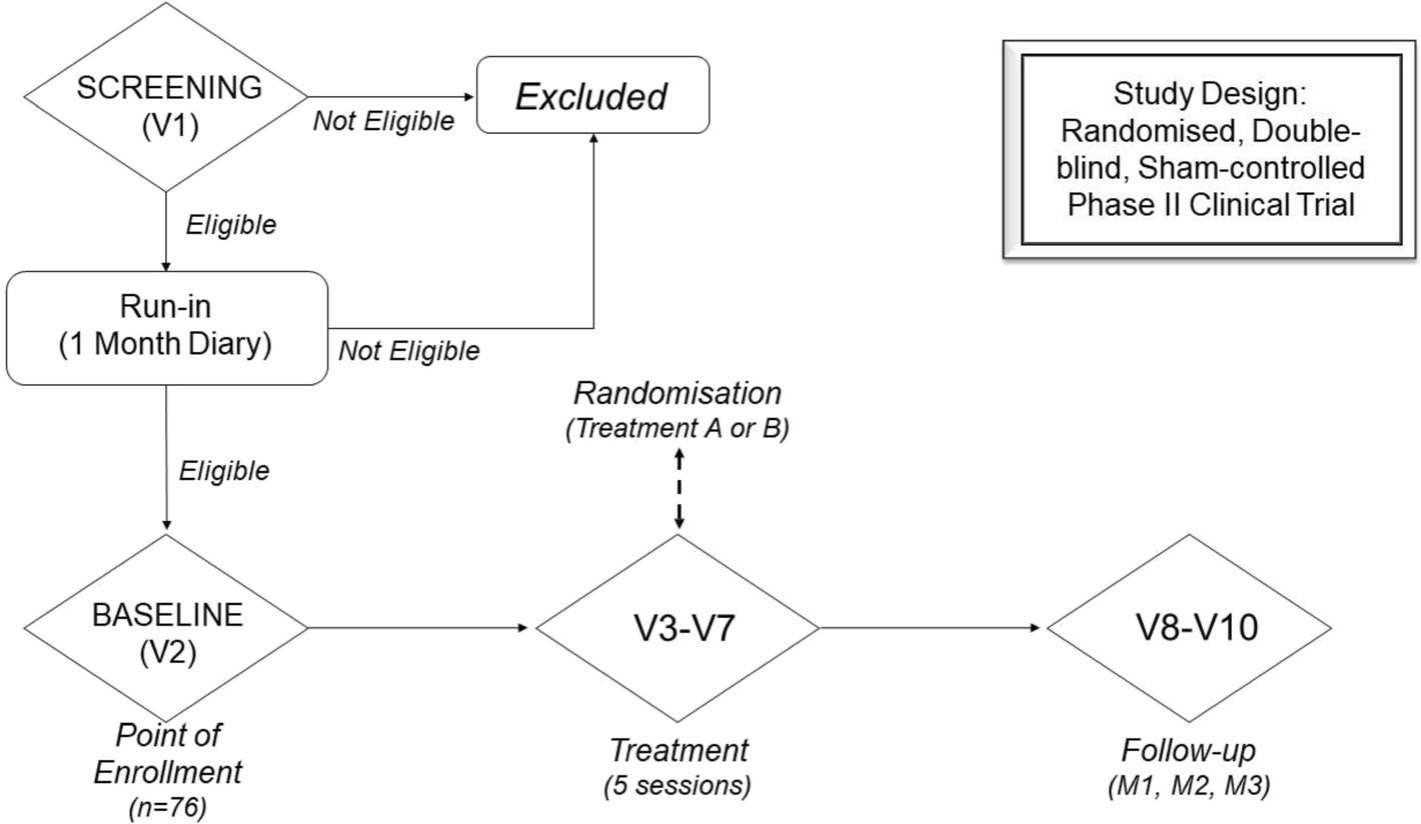
The overall design of the study. V, visit; M, month

Participants will be screened for eligibility during their first visit. Their history will be taken to establish the diagnosis according to ICHD-3. If they are eligible, they will be requested to record their headache attack for a month in the provided diary. Later, participants will receive rTMS treatment for a total of 5 sessions within 2 weeks. Only one treatment session will be given per day. Participants will receive 3 consecutive sessions per week in the 1st week and 2 consecutive sessions in the 2nd week as shown in Table 1. The follow-ups will be conducted at 1 month, 2 months and 3 months as shown in Fig. 1.

**Table 1:** Schedule of study

Study visit	V1	V2	V3	V4	V5	V6	V7	V8	V9	V10
Check eligibility	**X**	**X**								
Questionnaires		**X**								**X**
Study intervention			**X**	**X**	**X**	**X**	**X**			
Randomisation			**X**							
Laboratory test			**X**							**X**
Headache diary	**X**							**X**	**X**	**X**
AE monitoring			**X**	**X**	**X**	**X**	**X**	**X**	**X**	**X**
EEG		**X**								**X**
TCD		**X**								**X**

Questionnaires included are MIDAS, EQ-5D, PSQI, MSQv2.1, GPAQ, DASS21, FFQ and satisfaction measures of efficacy, tolerability, safety and expectations of r-TMS. Laboratory tests included are the measurement of serum serotonin level, serum beta-endorphin level and serum CGRP level

*AE* adverse events, *EEG* electroencephalography, *TCD* transcranial Doppler, *V* visit

### Sample size {14}

A statistical analysis was calculated according to the hypothesis testing method, which *α* = 0.05 and power = 80%. According to previous literature [13], the main outcome, the monthly headache days in rTMS treatment group, improved to 5.2 ± 4.9. Meanwhile, the monthly headache days in the sham treatment group improved to 8.9 ± 6.6. Thus, the initial sample size calculation is estimated based on the following formula.

$$ \mathrm{n}=2{\upsigma}^2{\left(\mathrm{Z}1\hbox{-} \upalpha +\mathrm{Z}1\hbox{-} \upbeta \right)}^2/{\left(\upmu 1\hbox{-} \upmu 2\right)}^2 $$

Where,

Z1-α/2 =1.96 at α=0.05

Z1-β =0.842 at 1-β = 0.80

σ^2^=4.9

Assuming a 30% attrition rate, a total of 76 patients will be needed to enrol in this study.

### Recruitment {15}

Participants are recruited from the nearby community, and they are encouraged to come to our Headache Research Clinic for the initial screening appointment. During the screening appointment, participants will be informed regarding the details of the study. A medical history will be taken to establish a headache diagnosis. Only participants who fit the criteria for migraine with aura or migraine without aura will be requested to record the headache diary.

## Assignment of interventions: allocation

### Sequence generation {16a}

A randomisation sequence will be generated with the help of online research randomiser (randomizer.org) by an external member, who is not directly involved in the study. The final code is only known to the external member, and the document will be stored in a secure locked safe by the external member. The key coding to the allocation will be revealed by the external member at the completion of the study.

### Concealment mechanism {16b}

To have a strict implementation of the generated random sequence, the concealed allocation is achieved using sequentially numbered, opaque and sealed envelopes (SNOSEs) prepared by an external member. An aluminium foil is kept inside the envelope to prevent any chances of deciphering. The envelopes will only contain the label of the devices which are “treatment A” or “treatment B”. This label will be put onto the sham coil and the rTMS coil by the same external member. This procedure prevents any influences either from the patients or the researchers towards the randomisation process.

### Implementation {16c}

The study number will be assigned at the point of enrollment after the patient signed and dated the informed consent. In this study, the study number is the same as the randomisation number; however, the point of randomisation is done directly before the first treatment session. The principal investigator and the blinded team may assign the participants to the intervention according to the treatment label inside the envelope.

## Assignment of interventions: blinding

### Who will be blinded {17a}

Participants, principal investigator and blinded team members including the data analyst are blinded to the assignment of the intervention. Any external members who will not directly be involved in the study will be in the unblinded group.

### Procedure for unblinding if needed {17b}

If any adverse events or pregnancy occurs for which knowledge of the identity of the test coil is necessary to manage the subject’s condition, the sealed emergency code key for that subject may be unblinded and the test coil will be identified immediately. The investigator will call the external member who generates the randomisation sequence and keeps it in a locked safe and request for the emergency code key for that subject to be broken to identify the test coil.

## Data collection and management

### Plans for assessment and collection of outcomes {18a}

The training sessions are done in a few sessions to ensure all the researchers know the trial procedures. Principal investigator which is a consultant neurologist had trained and assess each researcher who had been delegated to do the tasks. In addition, the questionnaires used in this trial are reliable and had been validated by other studies.

For data collection, patients who suffered from migraine will be recruited to attend Headache Research Clinic in Faculty of Medicine and Health Sciences, Universiti Putra Malaysia. Later, a screening appointment will be held in the clinic to diagnose the patients. Informed consent will be obtained from each migraine patients before they participate in this study.

### Plans to promote participant retention and complete follow-up {18b}

Participants will receive text messages and phone calls for scheduled visits (Table 1). At least two written attempts and one phone call will be made to follow-up patients before a patient is considered as lost to follow-up.

Participants’ data will be collected up until the point of dropping out. However, data collected from participants who do not complete the study will not be included in the analysis.

### Data management {19}

Data management will be conducted using appropriate database and validation programmes. Accurate and reliable data collection will be assured by verification and cross-check of the CRFs against the investigator’s records (source document verification). All collected data will be entered into a computer database and subjected to quality assurance procedures as dictated by Standard Operating Procedures of Malaysian GCP.

#### Data entry

All the data will be recorded into the computer programs using Microsoft Excel 2013.

#### Data validation and data query

Data will be abstracted retrospectively from computerised medical records by a database query for the identified patients. These data will be validated and augmented by abstracting data from the patients’ paper records.

#### Clean file and database lock

The missing data will be managed by running standard data-cleaning reports, which identify missing values or missing records. Once all the data collected during the visits have been transferred and captured in the database, cleaning, reconciliation and verification activities will be formed for smooth database lock.

Relevant bodies such as the institutional ethics committee (JKEUPM) and sponsor (RMC UPM) would also have access to the study data.

### Confidentiality {27}

All the information obtained in this study will be kept and handled in a confidential manner, in accordance with applicable laws and/or regulations. Subjects must be identified only by their assigned identification number and initial on all CRFs and other records and documents. When publishing or presenting the study results, the participant’s identity will not be revealed without his/her expressed consent. Individuals involved in this study and qualified monitors and auditors, the sponsor (UPM) or its affiliates and governmental or regulatory authorities may inspect and copy the medical records, where appropriate and necessary. Since this study will not reveal individual results, all the results will be kept confidential unless the subjects requested the result personally.

Biospecimens of the study participants may be tested in local university laboratories; however, the biospecimens will be coded, and information that can identify the participants will be removed.

### Plans for collection, laboratory evaluation and storage of biological specimens for genetic or molecular analysis in this trial/future use {33}

In this study, about 4–5 ml blood samples will be taken from the patients to measure serotonin level, calcitonin gene-related peptide level and beta-endorphin level in the serum during baseline and post-treatment. The blood will be taken from the antecubital vein of participants and will be collected in a serum separator blood tube.

The centrifugation process should be commenced within a relevant period and should not exceed more than 3 h to ensure accurate reading of the biochemical parameter. The extracted samples will be stored in a − 80 °C freezer until the samples are ready to be analysed. The biochemical parameters will be analysed using the commercial ELISA kit according to the manufacturer’s protocol.

## Statistical methods

### Statistical methods for primary and secondary outcomes {20a}

Data analysis will be performed by a medical statistician who is blinded to the entire allocation and treatment process. The SPSS statistical software package vision 22.0 will be used to assess the study data. The intention-to-treat principle will be used for all efficacy analyses. Two-tailed analyses will be performed, with a significant level set at 0.05.

Demographic characteristics and baseline measurement of the variables of each group will be summarised. Characteristics of the patients in each of the groups at baseline will be compared using independent *t* test or Mann-Whitney test for continuous variables, depending on the normality test for the variable. Chi-square or Fisher’s exact will be used to compare categorical variables between the groups.

The mean change of the monthly migraine days is the primary outcome measure of this study. For normality assessment, the Kolmogorov-Smirnov test and graphical approach through histogram with a normal curve will be used. Continuous variables will be presented as means ± SDs if they are normally distributed or as median with IQRs if they are skewed. For multivariate analysis, repeated measure analysis of variance (ANOVA) will be used to compare the between-subject effect (treatment effect), within-subject effect (time effect) and within-between-subject effect (treatment time effect) comparisons. Assumptions for the repeated measure ANOVA will be checked, which are assumptions of compound symmetry, normality of residuals and homogeneity of variance. Assumption of compound symmetry will be assessed through Mauchly’s test of sphericity, with the normality of residuals examined through histogram with overlaid normal curve of residuals, while homogeneity of variance will be assessed through Levene’s test. If one of the assumptions is not met, a proper remedial measure will be taken including extreme outliers’ elimination and data transformation.

The secondary outcome measures include the mean change of monthly migraine attacks, proportion of subjects with at least a 50% reduction, mean change of monthly pain intensity of migraine attacks, frequency and severity of adverse events in response to rTMS, mean changes in EEG and TCD, from baseline to endpoints in the study. All secondary outcome measures will be analysed following the same method for primary outcome measure analysis.

### Interim analyses {21b}

There will be no interim analysis in this study.

### Methods for additional analyses (e.g. subgroup analyses) {20b}

Comparison of the between-subject effects (treatment effect), within-subject effects (time effect) and within-between-subject effects (treatment time effect) will be done using ANOVA.

### Methods in analysis to handle protocol non-adherence and any statistical methods to handle missing data {20c}

As for missing data management, the last observation carried forward method will be used for the primary outcome.

### Plans to give access to the full protocol, participant-level data and statistical code {31c}

Not available.

## Oversight and monitoring

### Composition of the coordinating centre and trial steering committee {5d}

Not available.

### Composition of the data monitoring committee, its role and reporting structure {21a}

The progress report will be sent bi-annually to the sponsor and the ethics committee. The data monitoring committee from the sponsor will monitor the trial annually.

### Adverse event reporting and harms {22}

Information about all serious adverse events will be recorded on the Serious Adverse Event (SAE) page of the case report form. All events documented in the SAE Form must be reported within 24 h to the ethics committee and sponsor by fax. The investigator should not wait to receive additional information to fully document the SAE before notifying the ethics committee. A fax SAE form detailing relevant aspects of the SAE in question should follow telephone report of SAE. The investigator should also comply with the applicable regulatory requirements related to the reporting of unexpected serious reactions to the regulatory authorities.

Where applicable, information from relevant medical records and autopsy reports should be obtained. Any death or congenital abnormality, if brought to the attention of the investigator within 6 months after cessation of the study treatment, whether considered treatment-related or not, should be reported to the institutional ethics committee.

### Frequency and plans for auditing trial conduct {23}

The ethics committee (JKEUPM) will do the auditing process annually.

### Plans for communicating important protocol amendments to relevant parties (e.g. trial participants, ethical committees) {25}

Any amendment will be sent to the ethics committee (JKEUPM) again for ethical review. Any approved amendment will only be commenced after approval from the ethics committee. Any changes in the eligibility criteria will be informed to the participants during the re-consent procedure using the latest patient information sheet and consent form.

## Dissemination plans {31a}

The study outcome will be disseminated through peer-reviewed publications.

## Discussion

Although TMS is widely considered to be a safe technique, it has induced brief seizures in a small number of individuals worldwide [4, 9, 14]. Since 1998, seizures due to TMS have occurred, but mostly in studies operating outside the previously defined safe limits. Incidents of seizures in studies operating within the safe parameters have occurred in subjects using pro-epileptogenic medication. Considering the very large number of subjects who have participated in TMS studies since 1998 and the small number of seizures, the risk of TMS inducing seizures is considered to be very low [4]. A systematic review that included 93 sham-controlled RCTs [15] reported that headache or discomfort at stimulation site was the most commonly reported in both the active treatment and sham group (19.7% vs 10.1%, respectively). The second most reported adverse effect was dizziness accounting about 1.8% in the sham group and 2.8% in the active TMS group.

Previous research studies about depression had targeted DLPFC and found that high-frequency rTMS treatment could revert DLPFC activation to quite the normal level as shown using PET imaging and magnetic resonance [16]. Furthermore, another study using high-frequency rTMS targeting the same cortex had shown to have a therapeutic effect in chronic migraine patients [10]. In a study using capsaicin-induced pain on the dorsum of both hands, stimulation on the left DLPFC was noted to reduce the pain while stimulation on the right DLPFC gave no such effect, suggesting that the left DLPFC may have bilateral pain control. It was also observed that the high-frequency stimulation on the left DLPFC was able to restore the motor cortical excitability [17]. Considering the evidence, we decided to target DLPFC in our study.

In a randomised, sham-controlled study done on 100 migraine patients, 3 sessions of rTMS were given on alternate days [13]. The intensity was set at 70% RMT with 10-Hz frequency and 600 total pulses. The primary outcome of this study had shown that the headache frequency reduction was more in the rTMS group compared to the sham group. In terms of safety, there were no reported side effects in the sham group. In the rTMS group, only one patient had complained of drowsiness for 12 h and fortunately had no drowsiness when rTMS treatment was repeated after 1 month. Evidently, 3 cumulative sessions of high-frequency rTMS had able to successfully reduce migraine frequency.

Following the updated guideline of TMS in research and clinical settings [4], we designed a study protocol to treat episodic migraine in a preventive treatment setting. After carefully considering all factors including study feasibility, outpatient setting, local culture and community recruitment, we set the treatment sessions to only five sessions given in alternate weeks. We want to determine whether a low number of sessions given in a higher frequency would be able to ameliorate episodic migraine or not. On top of that, the primary outcome measure will be examined at 1, 2 and 3 months after treatment [10] to observe the stability of treatment effects as compared to the sham treatment over the 3-month period.

## Trial status

This is version 4 protocol dated 10 January 2019. The recruitment had begun on 15 April 2019 and the trial is ongoing.

## Abbreviations

CGRP: Calcitonin gene-related peptide
DASS21: Depression Anxiety Stress Scale 21
DLPFC: Dorsolateral prefrontal cortex
EEG: Electroencephalography
EQ-5D: European Quality-of-Life 5 Dimension Questionnaire
FFQ: Food Frequency Questionnaire
GCP: Good Clinical Practice
GPAQ: Global Physical Activity Questionnaire
MIDAS: Migraine Disability Assessment
MSQ 2.1: Migraine-Specific Quality of Life Questionnaire version 2.1
PSQI: Pittsburgh Sleep Quality Index
rTMS: Repetitive transcranial magnetic stimulation
SAE: Serious adverse events
TCD: Transcranial Doppler
